# Drug delivery in a tumour cord model: a computational simulation

**DOI:** 10.1098/rsos.170014

**Published:** 2017-05-24

**Authors:** M. E. Hubbard, M. Jove, P. M. Loadman, R. M. Phillips, C. J. Twelves, S. W. Smye

**Affiliations:** 1 School of Mathematical Sciences, The University of Nottingham, University Park, Nottingham NG7 2RD, UK; 2 Department of Medical Oncology, Leeds Teaching Hospitals NHS Trust, University of Leeds, St James’s University Hospital, Leeds LS9 7TF, UK; 3 Leeds Institute of Cancer and Pathology, University of Leeds, St James’s University Hospital, Leeds LS9 7TF, UK; 4 Institute of Cancer Therapeutics, University of Bradford, Bradford BD7 1DP, UK; 5 School of Applied Sciences, University of Hudderfield, Queensgate, Huddersfield HD1 3DH, UK; 6 Academic Division of Medical Physics, University of Leeds, Leeds LS2 9JT, UK

**Keywords:** computational modelling, mathematical modelling, drug delivery, drug transport and binding, pharmacokinetic resistance

## Abstract

The tumour vasculature and microenvironment is complex and heterogeneous, contributing to reduced delivery of cancer drugs to the tumour. We have developed an *in silico* model of drug transport in a tumour cord to explore the effect of different drug regimes over a 72 h period and how changes in pharmacokinetic parameters affect tumour exposure to the cytotoxic drug doxorubicin. We used the model to describe the radial and axial distribution of drug in the tumour cord as a function of changes in the transport rate across the cell membrane, blood vessel and intercellular permeability, flow rate, and the binding and unbinding ratio of drug within the cancer cells. We explored how changes in these parameters may affect cellular exposure to drug. The model demonstrates the extent to which distance from the supplying vessel influences drug levels and the effect of dosing schedule in relation to saturation of drug-binding sites. It also shows the likely impact on drug distribution of the aberrant vasculature seen within tumours. The model can be adapted for other drugs and extended to include other parameters. The analysis confirms that computational models can play a role in understanding novel cancer therapies to optimize drug administration and delivery.

## 1. Introduction

Resistance to systemic cancer drug treatment is recognized as a major limitation, not only of cytotoxic chemotherapy but also of targeted therapies, including tyrosine kinase inhibitors and monoclonal antibodies. The resistance of cells to particular drugs can be the result of intrinsic cell properties (primary resistance) or an adaptive response of the cells to exposure to the drug (secondary resistance). Different molecular mechanisms are involved in primary and secondary resistance, but a third class of resistance, pharmacokinetic (PK) resistance, has also been recognized and is relevant to both cytotoxic and targeted therapies [1,2]. This can be thought of as failure to deliver sufficient drug for long enough to induce a therapeutic response.

Pharmacokinetic drug resistance can be caused by the failure of a drug to penetrate throughout the tumour owing to physiological barriers imposed by the biology of the tumour [1]. The tumour vasculature and microenvironment is complex and heterogeneous, and contributes significantly to the PK resistance of the cancer cell [3]. Drug delivery is impaired principally because tumour vessels are distinct from normal tissue capillary networks in that they are poorly organized, inefficient and structurally different. These combine to generate regions of tumours that are poorly perfused with blood, leading to the generation of a hypoxic microenvironment and impaired drug delivery [1]. Furthermore, the presence of ‘leaky’ vessels, in conjunction with a more rigid extracellular matrix and dysfunctional fibroblasts, leads to elevated interstitial fluid pressure, impaired convective fluid flow and reduced drug penetration into the tumour [4]. Finally, it has also been argued that PK resistance can exercise a selection pressure on the tumour, accelerating the emergence of cellular clones resistant to the chemotherapeutic agent [5].

It is not only the characteristics of the tumour that limit drug penetration and distribution. The physicochemical characteristics of a drug and its circulating concentration, the plasma PKs, also play a key role in determining whether the drug reaches therapeutic concentrations throughout the cancer. Drug absorption (if given orally) and distribution within the patient depend on the drug’s chemical characteristics. Drug transport through membranes is affected by molecular size, polarity, pH, protein binding and the potential saturation of specific transporters. Patient characteristics, such as hepatic and renal function, will also influence drug metabolism and excretion. Drug dose, formulation, route and schedule of delivery will also impact upon plasma PKs, intra-tumoural drug concentrations and, ultimately, efficacy and toxicity.

Despite the potential importance of PK drug resistance, this is not a major focus of preclinical development or clinical trials. Indeed, remarkably little is known about intra-tumoural concentrations or distribution of most anticancer drugs and their impact, or otherwise, on efficacy and treatment outcome. There are a number of reasons for this paucity of data, including technical and logistical challenges, and pressure to reduce animal experimentation in drug development.

Computational models can play a key role in integrating and interpreting experimental and clinical PK and pharmacodynamic data, including determining the optimal schedule and dose, and response to therapy [6,7]. These computational models have the potential to reduce development time and cost, and to accelerate the drug development process. Optimal models need to be clinically relevant, encompass drug influx and efflux from the cell, include binding to intracellular structures, adapt to the PK profile as drug is delivered to the tumour and reflect the aberrant tumour vasculature.

We have focused on modelling intra-tumoural drug penetration to develop *in silico* models based on existing plasma PK data and experimental *in vitro* tissue penetration data. For the latter, we employed a transwell system and developed a model for the cytotoxic drug doxorubicin. We selected doxorubicin as it remains highly relevant clinically, its PKs have been well characterized and assays are amenable to this sort of work; there was good agreement between predicted and actual drug concentrations in this model [8]. Subsequently, we evaluated three computational models of doxorubicin transport through tumour cord geometry using parameters again based on parameterized *in vitro* experiments, varying the PK profile and binding affinity of drug to tumour cells [9]. One genuinely multidimensional model and two simplified, one-dimensional models, with radial symmetry assumed, gave similar results, and we were able to demonstrate the impact of altering the PK profile or binding on cell exposure to doxorubicin at arbitrary distances from a supplying blood vessel.

The aim of this paper is to modify the radially symmetric compartment model to allow for variations along the vessel supplying the drug (doxorubicin) to the tumour cord, to investigate the effect of altering: (i) the radius of the vessel, (ii) the permeability of the vessel wall, (iii) the flow velocity of the drug, and to (iv) explore the balance between drug binding and drug penetration, which is promoted by weaker drug binding. We explore a number of strategies with the potential to improve drug delivery throughout the tumour, including one suggested by Hauert *et al.* [10], in which we model the impact on tumour exposure to drug of repeat drug administration. The rationale for this approach is that binding sites in cells close to the vessel delivering drug will become saturated during the initial administration, enabling more drug to reach the more distant regions of the tumour during subsequent administrations.

In §2, we first outline the underlying binding model, which is parameterized by experimental data for doxorubicin, and then describe the full multidimensional tumour cord model and its discretization. Section 3 contains a summary of the three PK profiles we compare. In §4, a thorough comparison of the predicted effects owing to each of the PK profiles is presented, and the consequences of varying model parameters are investigated. These results are then summarized and discussed in §§5 and 6.

## 2. Models

The work in this paper builds on the one-dimensional, radially symmetric, compartment model we previously proposed [9]. Three distinct approximations of the radial variation in a longitudinally uniform tumour cord geometry were compared and we concluded that the quantitative and qualitative differences between the simulated results were small enough, relative to the uncertainty in the modelling process and in the experimental data that were used to parameterize the underlying binding processes, to select the simplest and fastest (referred to in [9] as the radially symmetric compartment model) as the most appropriate for predictive simulations.

In this paper, we retain the same binding model, which is recapitulated in §(a) for completeness, and recall that no explicit elimination or decay of drug is included beyond clearance via the central blood vessel which supplies the drug initially. This was assumed owing to the lack of functional lymphatics within tumour tissue, and the lack of data with which we might parameterize any additional generic clearance within living tissue. Because this omission is likely to render the model invalid for longer time periods, we limit our simulations to a maximum of 72 h.

In §(b), we generalize the radially symmetric compartment model of spatial variation of the drug concentration in a cylindrical tumour cord, centred on a single vessel supplying the drug [9], to allow for variation along the direction of the supplying vessel. The radial symmetry is retained, but the drug transport through the tissue is modelled in a two-dimensional plane, in the radial and axial coordinates. The advective transport of drug within this vessel is also modelled.

### 2.1. Binding model

The interaction of the chemotherapeutic agent with the microenvironment of cells is restricted to drug binding only, and described by a three-compartment model, composed of extracellular space (volume *V*_1_) with a concentration *C*_1_ of free drug and intracellular space (volume *V*_2_) with concentrations *C*_2_ and *C*_3_ corresponding to free and bound drug (where we understand the term bound to include both DNA-intercalated drug and drug bound to the cell in other ways). Binding is described by a simple kinetic model with drug binding reversibly to sites within the cell, which represents the principal form of binding for doxorubicin in living cells [11].

Applying the principle of mass action leads to three coupled ordinary differential equations which describe the system [9]:

2.1 $$\begin{array}{rl} V_{1}\frac{dC_{1}}{dt} & =ak_{1}(C_{2}-C_{1}), \end{array}$$

2.2 $$\begin{array}{rl} V_{2}\frac{dC_{2}}{dt} & =ak_{1}(C_{1}-C_{2})-V_{2}k_{2}C_{2}(C_{0}-C_{3})+V_{2}k_{-2}C_{3} \end{array}$$

**Figure 1.**
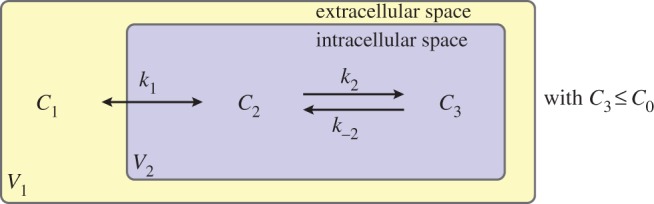
A three-compartment model of drug distribution in tissue. *C*_1_ represents extracellular drug concentration, *C*_2_ is free intracellular drug concentration and *C*_3_ is bound intracellular drug concentration.

2.3 $$\begin{array}{rl} \;\text{and}\;V_{2}\frac{dC_{3}}{dt} & =V_{2}k_{2}C_{2}(C_{0}-C_{3})-V_{2}k_{-2}C_{3}, \end{array}$$

in which *k*_1_ is the rate constant for the transmembrane transport of drug, *a* is the area of the interface between the extracellular and intracellular spaces (the surface area of the cells), *k*_2_ and *k*_−2_ are the drug binding and unbinding rates, respectively, and *C*_0_ is the concentration of binding sites within the cell. This model is illustrated by the schematic in figure 1. Values for the kinetic rate constants for the binding process, given in table 1, have been derived from a bespoke experimental binding assay, outlined in [9].

**Table 1.** Summary of model parameter values for baseline studies. (In the final column, ‘experiment’ refers to the fitting to experimental data described in [8,9], and ‘histology’ indicates estimation from histological tissue images, such as those illustrated at www.virtualpathology.leeds.ac.uk. The parameter *k*_0_ has been estimated based on the value of *r*_1_ (transport rate between cell layers) in the multilayer model of [8].)

variable	value	description	source of estimate
*l*	1.6×10^−5^ *m*	vessel radius	histology [12]
*L*_*r*_	1.96×10^−4^ *m*	cord radius (vessel + 9 cells)	histology [13,14]
*L*_*z*_	5.0×10^−4^ *m*	cord length (25 cells)	histology [15]
*r*	1.0×10^−5^ *m*	cell radius	histology [16]
*δ*	0.0625	extracellular : intracellular volume ratio parameter	histology [8,17]
*α*	1.94028×10^5^ *m*^−1^	membrane surface : tissue volume ratio	$\alpha =2/\left(r\sqrt{1+\delta }\right)$
*k*_0_	2.5×10^−6^ *m* *s*^−1^	permeability between cells	experiment [8]
*k*_1_	1.0×10^−6^ *m* *s*^−1^	permeability across cell membrane	experiment [9]
*k*_2_	0.90×10^−6^ μ*M*^−1^ *s*^−1^	drug binding rate	experiment [9]
*k*_−2_	14.0×10^−6^ *s*^−1^	drug unbinding rate	experiment [9]
*β*	140/9 μ*M*	unbinding/binding ratio	*β*=*k*_−2_/*k*_2_
*k*_*v*_	2.8×10^−6^ *m* *s*^−1^	permeability across vessel wall	estimate [18]
*D*	5.0×10^−11^ *m*^2^ *s*^−1^	interstitial diffusion rate	*D*=2*k*_0_*r*
*C*_0_	2.6×10^3^ μ*M*	binding site concentration	experiment [9]
*λ*	1.0×10^−3^ *m* *s*^−1^	flow velocity in vessel	estimate [19]

**Remark**

The kinetic rate parameters were derived under the assumption that the binding was reversible. In the limit of irreversible binding, i.e. the limit as $\beta =k_{-2}/k_{2}\to 0$ in equations (2.1)–(2.3), the mass-conserving steady-state solution is given by

2.4 $$\begin{array}{rl} C_{1}^{s} & =C_{2}^{s}\to \frac{\delta C_{1}(0)-C_{0}}{\delta +1}\;\text{and}\;C_{3}^{s}\to C_{0}\;\text{when}\;\delta C_{1}(0)\geq C_{0},\\ \;\text{and}\;C_{1}^{s} & =C_{2}^{s}\to 0\;\text{and}\;C_{3}^{s}\to \delta C_{1}(0)\;\text{when}\;\delta C_{1}(0)<C_{0}. \end{array}\}$$

in which *δ*=*V*_1_/*V*_2_ and *C*_1_(0) is the initial concentration of extracellular drug. In other words, the drug continues to bind until either all of the free drug is used up or the binding sites become saturated. These equations predict a piecewise linear dependence of the steady-state concentrations on *C*_1_(0), which is clearly not mirrored by the experimental data used to fit the model parameters in [9]. Hence, the data support the assumption that the binding is reversible when using this model to represent the dynamics.

### 2.2. Spatial model

Previously [9], the binding model was augmented with a spatial component by exploiting the shell-like nature of tumour cords, the geometric property that cells are broadly arranged in concentric cylindrical shells around a central blood vessel. In this work, a generalization of that one-dimensional model will be considered, which retains the cylindrical symmetry but allows variation in the axial direction as well as the radial direction. This enables the transport of drug along the vessel to be modelled as well as transport through the tissue.

In our two-dimensional model, we distinguish between the coordinate directions in the definition of the geometry by using subscripts *r* and *z* to represent, respectively, the radial and axial coordinates. In tissue, the spatial variation of the discrete drug concentrations are now given by

2.5 $$\begin{array}{rl} \delta_{1}V^{i}\frac{dC_{1}^{i,j}}{dt} & =A_{r}^{i-1/2}k_{0}(C_{1}^{i-1,j}-C_{1}^{i,j})+A_{r}^{i+1/2}k_{0}(C_{1}^{i+1,j}-C_{1}^{i,j})\\ & \;+A_{z}^{i}k_{0}(C_{1}^{i,j-1}-C_{1}^{i,j})+A_{z}^{i}k_{0}(C_{1}^{i,j+1}-C_{1}^{i,j})+a^{i}k_{1}(C_{2}^{i,j}-C_{1}^{i,j}), \end{array}$$

2.6 $$\begin{array}{rl} \delta_{2}V^{i}\frac{dC_{2}^{i,j}}{dt} & =a^{i}k_{1}(C_{1}^{i,j}-C_{2}^{i,j})-\delta_{2}V^{i}k_{2}C_{2}^{i,j}(C_{0}-C_{3}^{i,j})+\delta_{2}V^{i}k_{-2}C_{3}^{i,j} \end{array}$$

**Figure 2.**
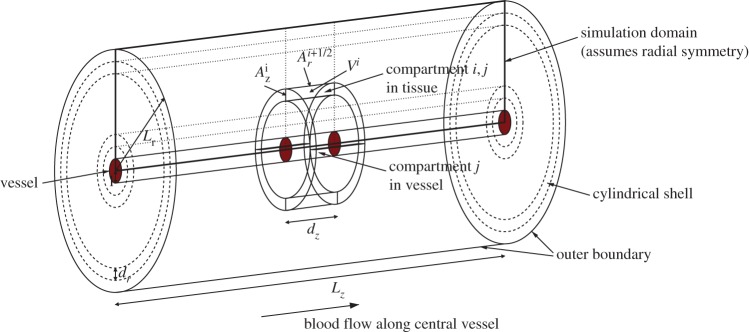
Geometry for a two-dimensional cylindrically symmetric compartment model. The rectangle with the thick outline represents the computational domain.

2.7 $$\begin{array}{rl} \;\text{and}\;\delta_{2}V^{i}\frac{dC_{3}^{i,j}}{dt} & =\delta_{2}V^{i}k_{2}C_{2}^{i,j}(C_{0}-C_{3}^{i,j})-\delta_{2}V^{i}k_{-2}C_{3}^{i,j}, \end{array}$$

for indices *i*=1,…,*n* and *j*=1,…,*m*, where *n* is the number of shells in the radial direction, *m* is the number of discs in the axial direction and *a*^*i*^ is the cellular surface area within any compartment in the *i*th shell: the volumes of these compartments are independent of the axial index *j*. The superscripts correspond, therefore, to the shell and disc indices of the discrete compartments, as illustrated by compartment *i*,*j* in figure 2. In this work, *n*=9 and *m*=25 are chosen with *d*=20 μ*m* so that each shell (and disc) can be approximately identified with a layer of biological cells. The geometry of the compartments is defined by

2.8 $$V^{i}=\pi ((2i-1)d_{r}+2l)d_{r}d_{z},\;A_{r}^{i+1/2}=2\pi (l+id_{r})d_{z},\;A_{z}^{i}=\pi ((2i-1)d_{r}+2l)d_{r},$$

in which *d*_*r*_=*d*_*z*_=*d* gives the dimensions of each compartment. Choosing *d*_*r*_=*d*_*z*_ means that the diffusive transport is isotropic when the permeability *k*_0_ is the same in both coordinate directions. Axial uniformity is assumed in the compartment geometry, so none of the local volumes or areas depends on the axial index, *j*. Note that the half-indices represent interfaces between shells.

At the vessel wall boundary the term $A_{r}^{1/2}k_{0}(C_{1}^{0,j}-C_{1}^{1,j})$ is replaced by $A_{r}^{1/2}k_{v}(C_{v}^{j}-C_{1}^{1,j})$, for *j*=1,…,*m*, where $C_{v}^{j}$ is the concentration of drug in the adjacent part of the supplying vessel. At the outer boundary in the radial direction, the term $A_{r}^{n+1/2}k_{0}(C_{1}^{n+1,j}-C_{1}^{n,j})$ is replaced with zero (also for *j*=1,…,*m*). A no-flux boundary condition is also applied at the boundaries in the axial direction, where the terms $A_{z}^{i}k_{0}(C_{1}^{i,0}-C_{1}^{i,1})$ and $A_{z}^{i}k_{0}(C_{1}^{i,m+1}-C_{1}^{i,m})$ are both replaced with zero along the whole boundary, i.e. for *i*=1,…,*n*.

The drug concentration in the one-dimensional representation of the vessel, denoted here by *C*_*v*_, is modelled using advective transport. This is coupled with the tissue by a term that allows for passage of drug through the vessel wall into the adjacent layer of cells, and we do not model the plasma binding as a separate compartment. The variation along the vessel is, therefore, governed by

2.9 $$V_{v}^{j}\frac{dC_{v}^{j}}{dt}=-A_{v}^{j}\lambda (C_{v}^{j}-C_{v}^{j-1})+A_{r}^{1/2}k_{v}(C_{1}^{1,j}-C_{v}^{j})$$

for *j*=1,…,*m*, where the index *j* corresponds to the axial index in the adjacent tissue compartment and $C_{v}^{0}=C_{v}(t)$ is the predefined PK profile in the blood vessel imposed at inflow, i.e. when *j*=1. A blood velocity *λ* drives flow along the vessel and no boundary condition is required at the outflow end of the vessel segment. The geometry is defined by

2.10 $$V_{v}^{j}=\pi l^{2}d_{z},\;A_{v}^{j}=\pi l^{2},$$

where *d*_*z*_=*d* is the width of a disc in the axial direction. The full geometry of the two-dimensional approximation is illustrated in figure 2, in which the vessel is represented by the central cylinder, and the values for the geometric and transport parameters are given in table 1.

## 3. Pharmacokinetic profiles

The clinical PKs of doxorubicin are well characterized in the literature [20]. Doxorubicin concentrations decay in a tri-exponential manner following intravenous (IV) bolus or infusion and typical parameters are available in the literature [21], in which doxorubicin is administered as an IV bolus. Clearance of the drug is modelled via the time dependence of this concentration profile.

The first PK profile considered here, subsequently denoted as PK1, is based on the data [21], in which *C*_*v*_(*t*) is assumed to decay as a tri-exponential (see also [22]) after a short infusion, i.e.:

3.1 $$C_{v}^{\mathrm{PK}1}(t)=\left\{ \begin{array}{ll} 0 & t\leq 0\\ \frac{D_{0}}{\tau }\left\{\frac{A}{A^{\prime }}(1-e^{-A^{\prime }t})+\frac{B}{B^{\prime }}(1-e^{-B^{\prime }t})+\frac{C}{C^{\prime }}(1-e^{-C^{\prime }t})\right\} & 0<t\leq \tau \\ \frac{D_{0}}{\tau }\left\{\frac{A}{A^{\prime }}(e^{A^{\prime }\tau }-1)e^{-A^{\prime }t}+\frac{B}{B^{\prime }}(e^{B^{\prime }\tau }-1)e^{-B^{\prime }t}+\frac{C}{C^{\prime }}(e^{C^{\prime }\tau }-1)e^{-C^{\prime }t}\right\} & t>\tau , \end{array}\right.$$

where *τ* is the infusion time, *D*_0_ is the dose and parameters *A*, *B*, *C*, *A*′, *B*′ and *C*′ are estimated by taking averages of the values given in table 2 of [21]. This gives (to three significant figures)

$$\begin{array}{rl} A & =7.46\times 10^{-2}\;l^{-1},\;A^{\prime }=2.69\times 10^{-3}\;s^{-1},\\ B & =2.49\times 10^{-3}\;l^{-1},\;B^{\prime }=2.83\times 10^{-4}\;s^{-1}\\ \;\mathrm{and}\;C & =5.52\times 10^{-4}\;l^{-1},\;C^{\prime }=1.18\times 10^{-5}\;s^{-1}. \end{array}$$

The duration of the perfusion for the total dose injected, *τ*=180 *s*, was also taken from Robert *et al.* [21] and the total dose *D*_0_=1.19827×10^2^ μ*mol* was calculated to give an ‘area under curve’ (AUC) of

3.2 $$\mathrm{AUC}\equiv \int_{0}^{\infty }C_{v}(t)\;dt=10^{4}\;\text{μ}M\;s\approx 2.78\;\text{μ}M\;h,$$

which is typical of what one might find in a patient [23,24]. The AUC is considered to reflect the actual tumour (i.e. cellular) exposure to drug, and to correlate with toxicity, i.e. to a lesser extent with clinical efficacy [25].

Two further PK profiles, both constructed to achieve the same AUC, are also considered in this work:

— a profile, denoted by PK2, in which there are three short infusions (to mimic the repeat drug administration suggested in [10]), each of one-third the dose of $C_{v}^{\mathrm{PK}1}(t)$, delivered at 24 h intervals, i.e.:
  3.3 $$C_{v}^{\mathrm{PK}2}(t)=\frac{1}{3}(C_{v}^{\mathrm{PK}1}(t)+C_{v}^{\mathrm{PK}1}(t-24\times 60\times 60)+C_{v}^{\mathrm{PK}1}(t-48\times 60\times 60)).$$
— a uniform profile, denoted by PK3, representing prolonged exposure at constant concentration over a period of 3 h, given by
  3.4 $$C_{v}^{\mathrm{PK}3}(t)=\left\{ \begin{array}{ll} 0.926 & 0<t\leq 10\;800,\\ 0 & \text{otherwise}. \end{array}\right.$$

These three profiles and the accumulation of their AUCs over a period of 72 h are illustrated in the electronic supplementary material, figures S1 and S2.

## 4. Results

Numerical experiments were carried out to investigate the effects of changing the PK profile of the supplied drug (to imitate different modes of delivery) and the model parameters. The computational domain representing the tissue for each simulation consisted of *m*=25 compartments in the axial direction and *n*=9 compartments in the radial direction (where each compartment has been chosen to be approximately the size of a biological cell) and was coupled with a vessel which also consisted of 25 compartments in the axial direction. Equations (2.5)–(2.7) and (2.9) were evolved in time using the Matlab function ode15s.

### 4.1. Varying vessel properties

**Figure 3.**
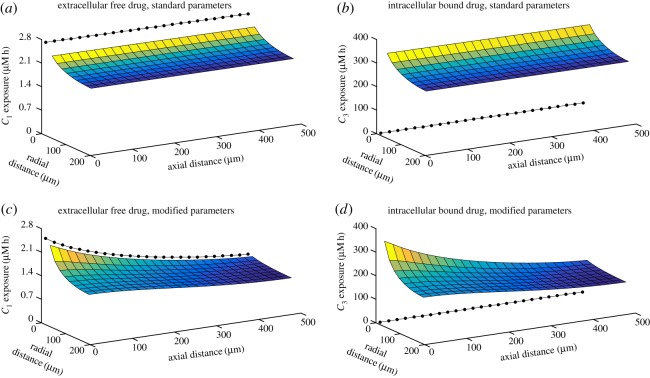
Spatialvariation of exposure (∫∗ dt) to extracellular drug, *C*_1_ (*a*,*c*), and bound drug, *C*_3_ (*b*,*d*), both at *t*=72 *h*. The black circles represent ‘exposure’ in the vessel and the surface plots represent exposure in the tissue. Each surface is coloured according to its height. Parameter values are as in table 1 (standard vasculature) for the top two plots, but modified so that $k_{v}\to 10\;k_{v}$, λ→λ/10 and l→l/2 (narrow, leaky vessels) for the bottom two plots. The single short-infusion pharmacokinetic profile, PK1, was used as input.

Figure 3 illustrates the spatial variation of the free extracellular (*C*_1_) and bound intracellular (*C*_3_) exposure profiles at 72 h for two sample simulations. The top plots show the results obtained for the parameters given in table 1; exposure decreases away from the vessel, but changes in the axial direction are hardly visible over the 500 μ*m* length of the simulated domain. The values of the tissue binding and transport parameters, *k*_0_, *k*_1_, *k*_2_, *k*_−2_ and *C*_0_, have been estimated for specific cancer cell lines [8,9], but the vessel-related parameter values, *k*_*v*_, *l* and *λ*, are likely to be highly variable in the leaky, chaotic, vasculature that is characteristic of cancerous tissue. Figure 3*c*,*d* illustrates how this might affect the spatial variation of the exposure by simulating a narrower vessel, with a less obstructive wall and slower flow; variation in the axial direction is now visible for this particular choice of modified vasculature, suggesting a higher risk of pharmacokinetic resistance owing to the non-uniformity of the distribution of drug.

A more thorough examination of the effects of changing parameters is described in the next section. Each model parameter was varied independently, with all remaining parameters retaining the values given in table 1. All simulations were run to $t_{\max}=72\;h$ and the output predicted by the model is illustrated using total exposure to bound drug at specific points in the tissue, $\int_{0}^{t_{\max}}C_{3}({\text{x}}^{i,j},t)\;dt$. We treat this as a determinant of the effect of the drug, in that increasing the exposure to bound drug of a cell should signify an increased likelihood of efficacy (and/or toxicity).

### 4.2. Varying model parameters

**Figure 4.**
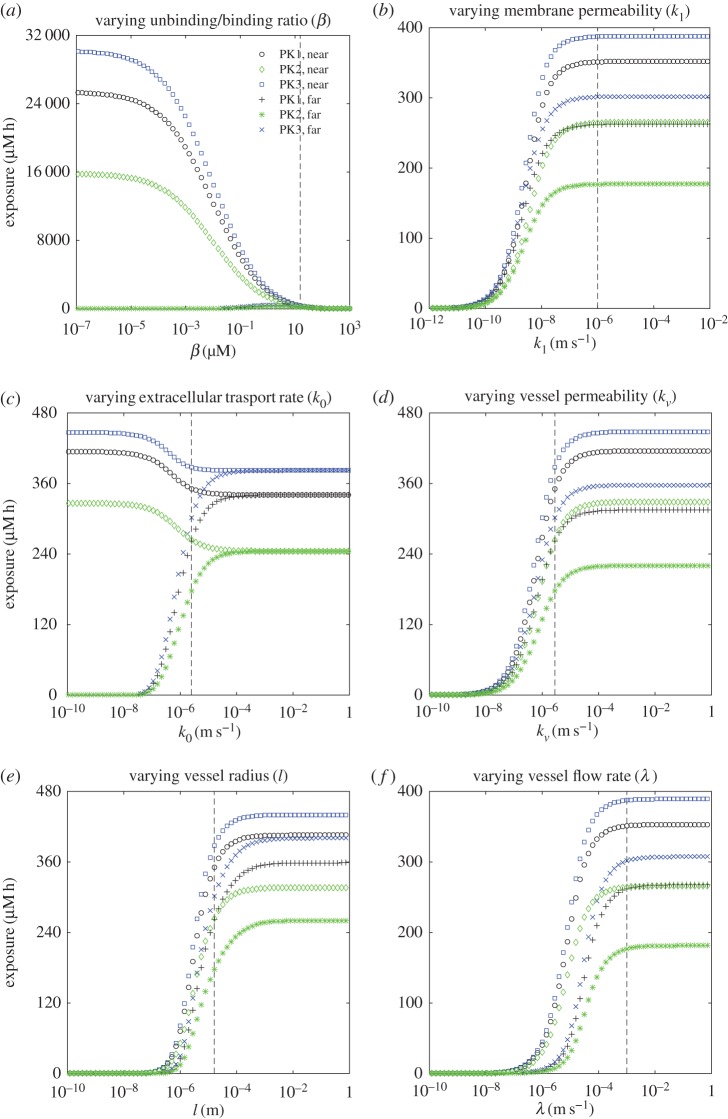
(*a*–*f*) Dependence of exposure to bound drug ($\int C_{3}\;dt$) at *t*=72 *h* on model parameters, for the two-dimensional, cylindrically symmetric model: each plot shows the response for all three PK profiles at points ‘near’ to the supply (*r*=*l*+10 μ*m*, *z*=10 μ*m*) and ‘far’ from the supply (*r*= *l*+170 μ*m*, *z*=490 μ*m*). The vertical dashed lines indicate the standard parameter values in table 1.

Figure 4 demonstrates how the exposure to bound drug at two points in the tissue varies as individual model parameters are changed. In each plot, the exposure is sampled at **x**^1,1^, the compartment closest to the inflow end of the supplying vessel, and **x**^*n*,*m*^, the compartment furthest from the supply of drug (which is most likely to exhibit PK resistance). Curves are shown for each of the three PK profiles defined in §3. The model output leads to the following observations which are characteristic of all simulation results obtained.

**Figure 5.**
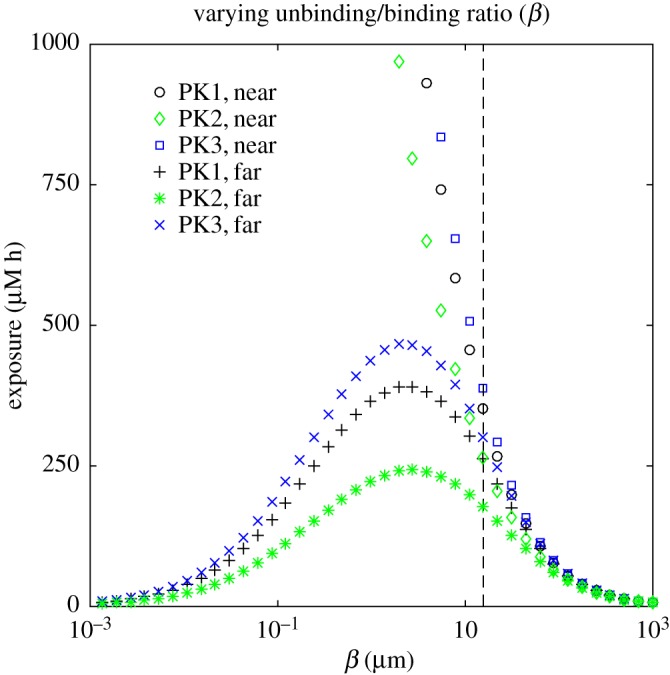
Dependence of exposure to bound drug ($\int C_{3}\;dt$) at *t*=72 *h* on binding ratio *β* for the two-dimensional, cylindrically symmetric model: the plot shows a magnified version of the bottom right corner of figure 4*a*.

**Figure 6.**
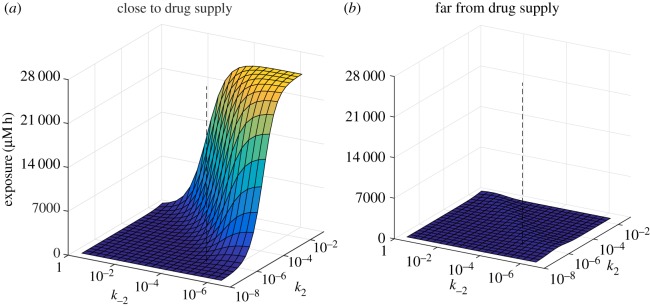
Dependence of exposure to bound drug ($\int C_{3}\;dt$) at *t*=72 *h* on binding rate *k*_2_, and unbinding rate *k*_−2_, for the two-dimensional, cylindrically symmetric model with profile PK1: close to the supplying vessel (*r*=26 μ*m*, *z*= 10 μ*m*, *a*); far from the supplying vessel (*r*=186 μ*m*, *z*= 490 μ*m*, *b*). Each surface is coloured according to its height. The vertical dashed lines indicate the standard values of *k*_2_ and *k*_−2_ in table 1.
  Figure 6 also shows that (except for very weak unbinding) the exposure depends on the ratio, *β*, of binding to unbinding rates and not the separate values of *k*_2_ and *k*_−2_. This observation is supported by the mathematical analysis in appendix A, which shows that, in the quasi-one-dimensional case where there is no variation in the axial direction
  4.4 $$\frac{C_{0}-\max_{t}C_{3}^{i}}{\beta }\int_{0}^{\infty }C_{v}(t)\;dt\leq \int_{0}^{\infty }C_{3}^{i}\;dt\leq \frac{C_{0}}{\beta }\int_{0}^{\infty }C_{v}(t)\;dt\;\forall i.$$
  This does not necessarily hold for small values of *β* in the fully two-dimensional case because strong binding causes the free drug concentration in the vessel to vary significantly in the axial direction.
— Exposure increases with *k*_1_, the rate of transport across the cell membrane. However, the value for *k*_1_ given in table 1 (indicated by the vertical dashed line) is in a region of parameter space where the exposure is insensitive to its value. This confirms the observation made when fitting the rate parameters to experimental data in [9], in which the value of *k*_1_ was chosen to be close to the minimum that could be taken without significantly changing the fit to the experimental assay data.
— When the diffusive transport rate in the tissue (represented here by the permeability *k*_0_) is varied, the effect on exposure depends on the distance from the supplying vessel. Close to the vessel the exposure actually decreases as the diffusion rate increases because, when transport is obstructed, the drug accumulates close to the vessel. Since it inhibits drug from moving away from the vessel, a low diffusion rate corresponds to low exposure further from the vessel. When the diffusion rate is high enough, the drug is uniformly distributed through the modelled region of tissue, so the exposure is the same throughout.
— Increasing *k*_*v*_, the permeability of the vessel, increases the exposure throughout the tissue. Very similar behaviour is seen when *l*, the radius of the supplying vessel, or *λ*, the velocity of flow in the vessel, is increased.

— The exposure of tissue to bound drug far from the supply is invariably lower than that close to the supply.
— The exposure due to a single short infusion (PK1) is lower than that due to a uniform profile (PK3) with the same AUC, typically by between 5 and 20%. The profile with three short infusions (PK2) gives even lower exposures. We emphasize here that the simulations are run to 72 h and that neither of the short infusion profiles delivers its full AUC within this timescale (as illustrated in the electronic supplementary material, figure S2).
  When the drug concentration throughout the vessel is held at the source concentration, *C*_*v*_(*t*), instead of modelling its escape into the surrounding tissue using equation (2.9), there is no variation in the axial direction and the concentration in the tissue depends only on the radial coordinate, indexed by *i*. This situation is closely approximated when simulations are run with the parameters given in table 1, as illustrated in figure 3*a*,*b*. In this case, as long as the prescribed, time-dependent profile satisfies $C_{v}(t)\to 0$ as t→0, it can be shown (see appendix A) that
  4.1 $$\int_{0}^{\infty }C_{1}^{i}\;dt=\int_{0}^{\infty }C_{2}^{i}\;dt=\int_{0}^{\infty }C_{v}(t)\;dt=\mathrm{AUC}\;\forall i,$$
  in which *i* represents the radial index of the computational shell. In other words, the AUCs for the free drug compartments are not only independent of the shape of the supplied PK profile but also equal to the AUC of the supplied profile. For the bound drug
  4.2 $$\beta \int_{0}^{\infty }C_{3}^{i}\;dt+\int_{0}^{\infty }C_{2}^{i}C_{3}^{i}\;dt=C_{0}\int_{0}^{\infty }C_{v}(t)\;dt\;\forall i,$$
  so its AUC does vary in space and depends on the shape of the supplied profile. However, when the concentration of bound drug is far from saturating the available binding sites, i.e. when $C_{3}^{i}\ll C_{0}\;\forall i$, it follows that the AUC for the bound drug compartment only depends weakly on the shape of the supplied PK profile, because
  4.3 $$\int_{0}^{\infty }C_{3}^{i}\;dt\approx \frac{C_{0}}{\beta }\int_{0}^{\infty }C_{v}(t)\;dt=\frac{C_{0}}{\beta }\mathrm{AUC}\;\forall i.$$
— Close to the vessel, drug exposure increases as *β*=*k*_−2_/*k*_2_ decreases, i.e. with stronger binding and/or weaker unbinding. However, at greater distances from the vessel there is an optimal value of *β*, below which the exposure of the tissue to bound drug decreases. This is because, when binding is relatively strong, the drug is captured by the cells close to the vessel and not transported to the more distant cells.
  The value of *β* given in table 1 (indicated by the vertical dashed line in the figure) gives a very low exposure, relative to what might be achieved close to the vessel with stronger binding. However, it also gives a fairly uniform distribution through the tissue (figure 3*b*), so all regions of the tissue receive similar amounts of drug. In fact, the exposure furthest from the supply is close to the maximum achievable with the other parameters fixed. This is more clearly visible in the magnified plot shown in figure 5, and in figure 6*b*, where a ridge indicates the values of the binding rate *k*_2_, and the unbinding rate *k*_−2_, for which exposure at a distance from the supply is maximized.

### 4.3. Varying dose

**Figure 7.**
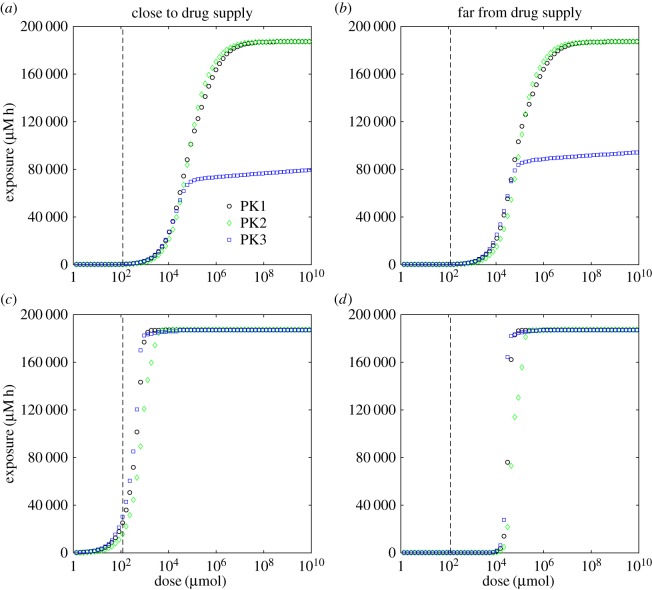
Dependence of exposure to bound drug ($\int C_{3}\;dt$) at *t*=72 *h* on dose, *D*_0_, for the two-dimensional, cylindrically symmetric model: close to the supplying vessel (*r*=26 μ*m*, *z*=10 μ*m*; *a*,*c*); far from the supplying vessel (*r*=186 μ*m*, *z*=490 μ*m*; *b*,*d*). In one set of simulations, the binding rate *k*_2_, and unbinding rate *k*_−2_, are the standard values taken from table 1 (top), in the other, much stronger binding and weaker unbinding are used (*k*_2_=2.95×10^−2^ μ*M*^−1^ *s*^−1^ and $k_{-2}=4.38\times 10^{-7}\;s^{-1}$, bottom). The vertical dashed lines indicate the value of *D*_0_ in §3.

In this paper, we choose predominantly to present the model predictions in terms of exposure to bound drug, treating it as an indicator of treatment efficacy. Having investigated its dependence on model parameters, we now analyse the relationship of drug exposure with the dose of drug, *D*_0_ in equation (3.1) of §3, for each of the PK profiles presented in that section. Figure 7 shows the dependence of exposure on dose at positions close to and far from the drug supply.

**Figure 8.**
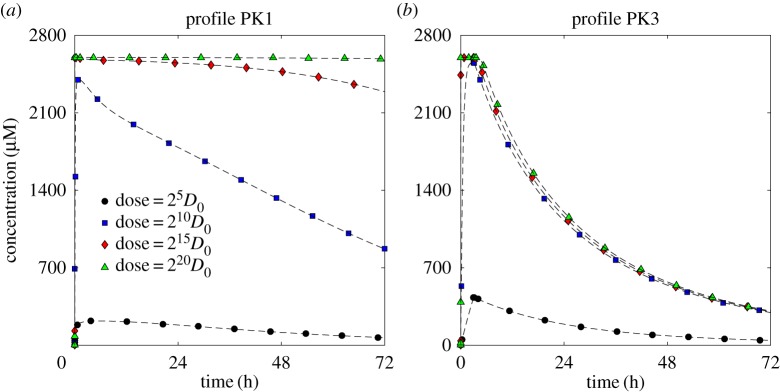
Comparison of bound drug, *C*_3_, profiles for different total doses ($\int_{0}^{\infty }C_{v}(t)\;dt$): single, short infusion (PK1, *a*) and prolonged subjection to constant concentration (PK3, *b*).

— For low doses, all three PK profiles give very similar exposures, even though the time variation of *C*_3_ is very different for each profile. The electronic supplementary material, figure S4, provides an example of how *C*_3_ can vary in time at different distances from the supply.
— For high doses, the uniform profile (PK3), representing prolonged subjection to a constant concentration, gives significantly lower exposure than the other two. The cause is illustrated in figure 8. For high doses the binding sites rapidly become saturated, i.e. *C*_3_≈*C*_0_: in all cases the uniform profile (PK3) drops to zero after 3 h, so the drug starts to return to the vessel, but the two short infusion profiles (PK1 and PK2) continue to supply drug at levels which maintain saturation for the full 72 h simulation time. It is clear from equation (4.2) that, even when variation is only allowed in the radial direction, the shape of the supply profile might have a significant effect on exposure when saturation is approached, though we acknowledge that profile PK3 has been chosen to represent a specific, exaggerated circumstance which might be created *in vitro* but is not physiologically realistic.
— The exposure to bound drug is fairly uniform throughout the computational domain for all doses when the parameter values from table 1 are used (e.g. figure 3*a*,*b*), so very little difference is seen between the exposures close to and far from the supply in figure 7*a*,*b*. However, when the values of the binding and unbinding rates, *k*_2_ and *k*_−2_, respectively, are replaced with those corresponding to the far right corner of the domain in figure 6 (an extreme situation, chosen to illustrate the consequences of a microenvironment which supports strong spatial heterogeneity in drug distribution), significant differences appear between different parts of the tissue, which are visible in figure 7*c*,*d*. The cells furthest from the drug supply now require a dose two orders of magnitude higher than cells adjacent to the supply if they are to receive the same exposure.
  It can also be seen in figure 7 that the exposures of all three PK profiles exhibit similar dependence on dose when the stronger binding is used. In this case, saturation at high doses is maintained for longer than with weaker binding when the uniform profile (PK3) is administered, so the exposures become close to those of both short infusion profiles. For the specific parameter values chosen here, the exposure due to the profile with three short infusions (PK2) is lowest, but this does not always hold, e.g. the profiles shown in figure 7*a*,*b* indicate that PK2 gives the greatest exposure of the three for high doses when the original set of parameters (table 1) is used.

**Remark**

**Figure 9.**
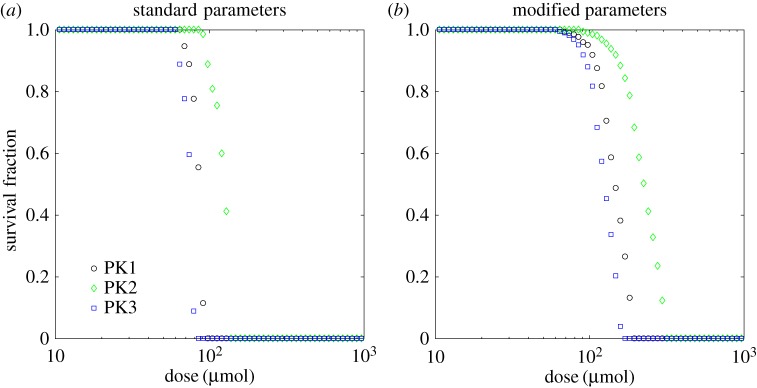
Dependence of survival fraction on dosefor the two-dimensional cylindrically symmetric model where a cell is assumed to die if its exposure to bound drug exceeds 165 μ*M* h: standard binding rate *k*_2_, and unbinding rate *k*_−2_, from table 1 (*a*); much stronger binding and weaker unbinding (*k*_2_=2.95×10^−2^ μ*M*^−1^ *s*^−1^ and $k_{-2}=4.38\times 10^{-7}\;s^{-1}$, *b*).

Instead of using exposure to bound drug as an indicator of the response to drug, it could be assumed that the damage done to a cell by the drug depends monotonically on its exposure to bound drug; an individual cell might survive if the damage (exposure) is below a particular threshold value or die if it exceeds that threshold. In models without spatial heterogeneity, the exposure could be converted into a survival fraction using, for example, a Hill equation [26]; we can imitate this by applying a threshold to the exposure to bound drug in each computational compartment (the sizes of which have been chosen to match the size of a biological cell). Applying the same threshold to every compartment is analogous to using a Heaviside function in place of the Hill equation—it represents the limit as the Hill exponent tends to infinity (and the higher the Hill exponent, the lower is the phenotypic heterogeneity of the population). In practice, we do not have the data to allow us to estimate a value for this threshold (and certainly not its variability between cells), so we provide figure 9 for illustrative purposes only. It shows the dependence of survival fraction, in the sense defined above, on dose for the two sets of parameters used to produce figure 7. A low survival threshold was chosen, for which the profile with three short infusions (PK2) clearly gives lower efficacy, which corresponds to the higher doses required to achieve this exposure threshold value. However, it can be seen from figure 7 that the order of effectiveness of the three PK profiles depends on both the threshold value and the choice of model parameters. An extreme example of this would be applying a threshold of 1.5×10^5^ μ*M* *h* when the parameters from table 1 are used: in this case three short infusions is the most effective mode of delivery and prolonged exposure at constant concentration kills no cells at all. Allowing the threshold to vary between cells would tend to smooth out the transition regions visible in figure 9.

### 4.4. Varying distance from source

To assess PK resistance for regions where the vasculature is more sparse, we consider a modified geometry in which an avascular tumour spheroid is surrounded by vascularized tissue in which the drug concentration is that of the specified PK profile. This geometry and the numerical approximation are described in detail in the electronic supplementary material, S1.3.

**Figure 10.**
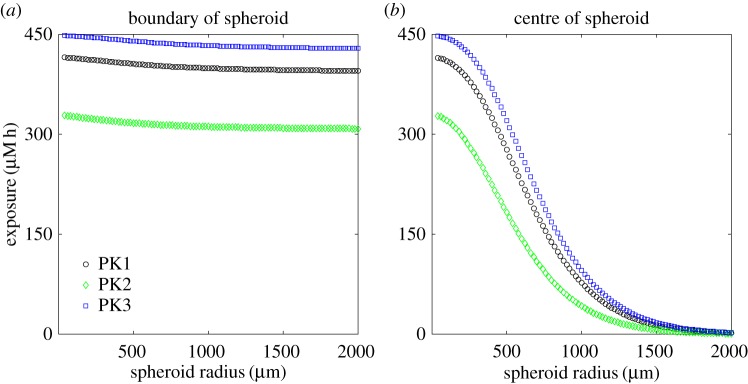
Dependence of the exposure to bound drug ($\int C_{3}\;dt$) at *t*=72 *h* on spheroid radius, for the one-dimensional, spherically symmetric model: at the edge of the spheroid (*a*) and in its centre (*b*). Each plot shows the exposure for all three PK profiles.

Figure 10 shows the dependence of the exposure to bound drug on the radius of the spheroid, sampling at both the edge and the centre of the spheroid. It can be seen that exposure close to the surface of the spheroid has very little dependence on the size of the spheroid. However, the exposure to bound drug of the centre of the spheroid is strongly influenced by its distance from the supply: for the parameter values given in table 1, the exposure at the centre of a 2 mm radius spheroid has dropped almost to zero. This region would typically also be nutrient-deficient and possibly necrotic.

## 5. Discussion

A model of drug transport in a tumour cord has been developed to explore the effect of different drug delivery schedules and drug-binding parameters on tumour exposure to doxorubicin over a period of up to 72 h. In particular, we have used the model to assess the benefits of sequential administration of drug doses and at steady state. In common with all computational approaches, the model makes a number of simplifications, including neglecting changes in drug clearance beyond that reflected by the concentration–time curve of drug levels in the central blood vessel. Model parameters have been determined *a priori* from a combination of *in vitro* experimental findings and the literature. Importantly, all model parameters (apart from the transport rate across the cell membrane, for which only a minimum value could be estimated from the experimental assay data) are within or close to the regions of parameter space in which the exposure is sensitive to their values (indicated by the proximity of the vertical dashed lines to the regions of the graphs in which exposure is changing rapidly in figures 4–6). Likewise, small modifications of some of the parameters, representing tumour vascularization, for example, can potentially have a significant impact on drug delivery and, therefore, certain drug combinations should be administered carefully. Subject to the caveats above, the model generates several relevant findings.

Exposure to bound drug is lower further away from the supply, as illustrated in figure 3. This is intuitively obvious, but mathematical analysis of the model (see appendix A) confirms further that exposure to bound drug is similar for all three PK profiles, except when saturation of binding sites is approached; at that point the uniform profile (prolonged subjection to a constant concentration) gives significantly lower exposures. This is likely to be true not only for tumours but also for healthy tissues in relation to toxicity. Interestingly, some clinical studies have suggested that continuous infusion of doxorubicin is associated with less cardiac toxicity than bolus administration [27,28]. This has been attributed to lower peak drug concentrations, but the model suggests that overall exposure may also be lower with prolonged infusion of doxorubicin. The effect of drug exposure on distance from the blood supply, shown in the context of a tumour spheroid in figure 10, demonstrates the challenge of delivering drug to the avascular, hypoxic core of a tumour. Such regions are recognized as being chemo-resistant and the model demonstrates the role of PK drug resistance in this context and offers the prospect of modifying the model parameters to improve delivery to such regions and guide the development of novel agents with the necessary characteristics [1,29,30,31].

From a therapeutic perspective, the most effective mode of administration depends on the threshold of exposure that must be exceeded to kill tumour cells (see figures 7 and 9), as well as the model parameters (figure 4). As the binding becomes stronger relative to the unbinding, exposure to bound drug increases close to the supply; further from the supply there is, however, an optimal binding ratio, above which the exposure to bound drug starts to decrease (shown in figure 4*a* and in figure 5). While we have not included an explicit model of cell-kill, this finding implies that drugs which bind avidly may have reduced clinical effectiveness, as they would fail to reach cells distant from the vessel. An example may be the monoclonal antibody trastuzumab, which binds to the cell surface HER2 receptor that is overexpressed in about 15% of breast cancers. Experiments in spheroids suggest that penetration of trastuzumab deeper into the spheroid may indeed be limited by binding to the superficial cell layers [32].

Exposure to bound drug also increases as: (i) the transport rate across the cell membrane increases; (ii) the permeability of the wall of the supplying vessel increases; (iii) the radius of the supplying vessel increases; and (iv) the velocity of the blood flow increases (as illustrated in figure 4). The tumour vasculature is recognized as being disorganized, with vessels that are more permeable, immature and tortuous, with inconsistent diameter and impaired blood flow [2]. Our model suggests that these characteristics of the tumour vasculature may have differing effects on drug exposure. ‘Normalizing’ the tumour vasculature, as has been shown with the microtubule-targeted cytotoxic eribulin, may enhance the delivery of anticancer drugs [33]. Likewise, some studies demonstrate that bevacizumab increases the number of small vessels and tumour perfusion, allowing a better distribution of paclitaxel when given in combination [34].

When diffusion through the tissue is very rapid, the distribution of drug is uniform (and so is the exposure to bound drug). When diffusion is slow, exposure to bound drug is high close to the supplying vessel but rapidly drops away from the vessel (figure 4*c*). The clinical significance of this observation is unclear but it is noteworthy that one of the explanations proffered for the very limited efficacy of chemotherapy in pancreatic cancer is the presence of extensive fibrosis, common within such cancers, that is postulated to affect drug delivery [35]. The dependence of uniformity of exposure on transport rate suggests that the raised interstitial pressure observed within tumours will indeed influence drug penetration and distribution in tumours [4]. Similarly, uniform exposure throughout the tissue (and hence good drug penetration) is facilitated by fast transport within the vessel, across the vessel wall and across the cell membrane (figure 4).

The ultimate aim of predictive modelling of the type presented here is the optimization of drug delivery, but this requires additional knowledge of the dependence of cell survival on the time course of drug concentration. To illustrate the behaviour of our model, we have chosen to indicate the local effectiveness of the treatment by the exposure of the tissue to bound drug. However, the toxicity of doxorubicin is not solely dependent on AUC, particularly at low concentrations, and there is some evidence that peak concentration might be a better indicator of cell-kill ([22,26] and references therein). It would be straightforward to conduct our study using either AUC or peak values of any of the concentrations in our model, but a better understanding of these dependencies would be required before the model could be deemed to be truly predictive, and therefore effective in aiding treatment optimization. We are also aware that, unlike the peak value, the AUC would continue to increase after the end of the 72 h time window investigated here. Although we believe that we would need a more realistic model of drug clearance for our model to be valid for longer times, we note that running our simulations beyond 72 h did not affect the qualitative behaviour of the results.

The model can be adapted to other cytotoxics or molecularly targeted cancer drugs and extended to include, for example, tumour heterogeneity and different geometries; it can also be applied to other clinical indications. Particularly attractive is the potential to incorporate the model in drug development to select agents most likely to achieve uniform distribution of drug at a relevant concentration throughout the target tissue or tumour. Changing the physico-chemical properties of the drug alters the associated parameters incorporated in the model, such as binding rates, but the values can be identified from drug-specific data. This may allow the identification, from a range of candidate molecules, of the one(s) most likely to address the issue of PK resistance. This could have important implications in terms of the time and expense of preclinical evaluation, as well as addressing the principles of the 3Rs (replacement, reduction and refinement) that have been developed as a framework for humane animal research and subsequently become embedded in guidelines and legislation regulating the use of animals in scientific procedures.

## 6. Conclusion

Our model of drug transport in a tumour cord explores the effect of different drug delivery schedules and tumour-binding parameters on tumour exposure to doxorubicin, using parameters determined from experimental data. While acknowledging that the model remains a simplification of a complex system, we believe that such computational models can play an important role in understanding existing and novel cancer therapeutics by generating quantitative and testable insights that inform further experiments in the clinically important area of optimizing drug delivery.

## Supplementary Material

Additional profiles and model description

## Data accessibility

All data can be accessed at the Dryad Digital Repository http://dx.doi.org/10.5061/dryad.j5s12 [36].

## Appendix A. Dependence of exposures on area under curve

We restrict ourselves here to the one-dimensional, cylindrically symmetric case (or, equivalently, the multidimensional model described in §(b) with $C_{v}^{j}=C_{v}(t)$ imposed in the vessel in place of using equation (2.9)). We, therefore, consider a model from which axial variation has been removed:

A 1 $$\begin{array}{rl} \delta_{1}V^{i}\frac{dC_{1}^{i}}{dt} & =A^{i-1/2}k_{0}(C_{1}^{i-1}-C_{1}^{i})+A^{i+1/2}k_{0}(C_{1}^{i+1}-C_{1}^{i})+a^{i}k_{1}(C_{2}^{i}-C_{1}^{i}), \end{array}$$

A 2 $$\begin{array}{rl} \delta_{2}V^{i}\frac{dC_{2}^{i}}{dt} & =a^{i}k_{1}(C_{1}^{i}-C_{2}^{i})-\delta_{2}V^{i}k_{2}C_{2}^{i}(C_{0}-C_{3}^{i})+\delta_{2}V^{i}k_{-2}C_{3}^{i} \end{array}$$

A 3 $$\begin{array}{rl} \;\text{and}\;\delta_{2}V^{i}\frac{dC_{3}^{i}}{dt} & =\delta_{2}V^{i}k_{2}C_{2}^{i}(C_{0}-C_{3}^{i})-\delta_{2}V^{i}k_{-2}C_{3}^{i}, \end{array}$$

for *i*=1,…,*n*, where *n* is the number of shells, and *a*^*i*^ is the cellular surface area within the *i*th shell. The superscript corresponds to the shell number, and this index increases with distance from the centre of the cylindrical coordinates. Half-indices correspond to interfaces between shells. At the inner boundary, a predefined PK profile, *C*_*v*_(*t*), is prescribed, and hence when *i*=1, the term $A^{1/2}k_{0}(C_{1}^{0}-C_{1}^{1})$ is replaced by $A^{1/2}k_{v}(C_{v}(t)-C_{1}^{1})$ in equation (A 1) (in which *k*_0_ has also been replaced by *k*_*v*_, the permeability of the vessel wall). A no-flux condition is imposed at the outer boundary of the tumour cord by replacing the term $A^{n+1/2}k_{0}(C_{1}^{n+1}-C_{1}^{n})$ with zero when *i*=*n* in equation (A 1). The precise values of the geometric quantities do not matter for the purposes of this proof.

Similar analysis can be applied directly to the spherically symmetric model used in §(d) and described fully in the electronic supplementary material, S1.3. However, the following arguments do not hold for the general two-dimensional model, in which equation (2.9) is used to model flow in the vessel, because the flux of drug at the outflow end of the central vessel is not known.

First note that each of equations (A 1)–(A 3) can be written in the form

A 4 $$\frac{dC_{k}^{i}}{dt}=f_{k}({\text{C}}_{k})-g_{k}({\text{C}}_{k})C_{k}^{i},$$

in which *i*=1,…,*n*, is the compartment index, *k*=1,2,3, is the drug component index, **C**_*l*_ is a vector containing all the values of $C_{l}^{i}$ *except* those for which *l*=*k*, and *f*_*k*_ and *g*_*k*_ are linear functions of these values with non-negative coefficients (as long as $C_{3}^{i}\leq C_{0}$, which is guaranteed in this case by the form of equation (A 3) and $C_{3}^{i}(0)=0$). As a consequence, since $C_{k}^{i}$ are all initially zero and *C*_*v*_(*t*)≥0, the $C_{k}^{i}$ must remain non-negative for all time.

Now, given any positive integer *n*, define the local and global masses for each of the drug compartments

$$M_{k}^{i}=V^{i}C_{k}^{i},\;M_{k}=\sum_{j=1}^{n}M_{k}^{j},\;\text{for}\;k=1,2,3,\;1\leq i\leq n,$$

so that summing equations (A 1)–(A 3) for all of the compartments and all of the species gives, after applying the zero-flux boundary conditions,

$$\delta_{1}\frac{dM_{1}}{dt}+\delta_{2}\frac{dM_{2}}{dt}+\delta_{2}\frac{dM_{3}}{dt}=A^{1/2}k_{v}(C_{v}(t)-C_{1}^{1}),$$

in which we have assumed zero flux through the boundary far from the vessel. Now, since $C_{v}(t)\to 0$ as t→∞, it must follow that $C_{1}^{1}\to 0$ as t→∞, otherwise (since, in this model, $C_{k}^{i}\geq 0,\;\forall i,k$ and for all time) there exist ϵ,T∈R such that $C_{1}^{1}\geq \epsilon >0$ for *t*>*T*, and the total mass of drug, *M*=*δ*_1_*M*_1_+*δ*_2_*M*_2_+*δ*_3_*M*_3_, would eventually become negative, which contradicts non-negativity of the concentrations. We can also define partial masses

$$P_{k}^{i}=\sum_{j=i}^{n}M_{k}^{j}\;\text{for}\;k=1,2,3,\;1\leq i\leq n,$$

for which it follows, from equations (A 1)–(A 3) and their zero-flux boundary conditions, that

$$\delta_{1}\frac{dP_{1}^{i}}{dt}+\delta_{2}\frac{dP_{2}^{i}}{dt}+\delta_{2}\frac{dP_{3}^{i}}{dt}=A^{i-1/2}k_{0}(C_{1}^{i-1}-C_{1}^{i})\;\text{for}\;2\leq i\leq n.$$

By a similar argument to that above, $C_{1}^{i-1}\to 0$ as t→∞ implies that $C_{1}^{i}\to 0$ as t→∞ for *i*=1,…,*n*, since the concentrations and masses must all remain non-negative. This argument can, therefore, be used repeatedly to show that $C_{1}^{i}\to 0$ as t→∞ for all *i*, whatever the value of *n*. Furthermore, since

$$\delta_{2}\frac{dM_{2}^{i}}{dt}+\delta_{2}\frac{dM_{3}^{i}}{dt}=a^{i}k_{1}(C_{1}^{i}-C_{2}^{i})\;\text{for}\;1\leq i\leq n,$$

it follows that $C_{2}^{i}\to 0$ as t→∞ for *i*=1,…,*n*. Using this knowledge, and the above argument a third time

$$\frac{dM_{3}^{i}}{dt}=V^{i}k_{2}C_{2}^{i}(C_{0}-C_{3}^{i})-V^{i}k_{-2}C_{3}^{i}\;\text{for}\;1\leq i\leq n,$$

implies that $C_{3}^{i}\to 0$ as t→∞ for *i*=1,…,*n*.

We now know that all of the compartment concentrations tend to zero as t→∞, so we can consider

$$\int_{0}^{\infty }\delta_{1}\frac{dM_{1}}{dt}+\delta_{2}\frac{dM_{2}}{dt}+\delta_{2}\frac{dM_{3}}{dt}\;dt=\int_{0}^{\infty }A^{1/2}k_{v}(C_{v}(t)-C_{1}^{1})\;dt.$$

The left-hand side of this equation is equal to zero, because the zero concentrations at t=0,∞ give

$${\left[\delta_{1}M_{1}+\delta_{2}M_{2}+\delta_{2}M_{3}\right]}_{0}^{\infty }=0.$$

It immediately follows that

$$\int_{0}^{\infty }C_{1}^{1}\;dt=\int_{0}^{\infty }C_{v}(t)\;dt.$$

By a similar argument,

$$0=\int_{0}^{\infty }\delta_{1}\frac{dP_{1}^{i}}{dt}+\delta_{2}\frac{dP_{2}^{i}}{dt}+\delta_{2}\frac{dP_{3}^{i}}{dt}\;dt=\int_{0}^{\infty }A^{i-1/2}k_{0}(C_{1}^{i-1}-C_{1}^{i})\;dt\;\text{for}\;2\leq i\leq n,$$

which implies that

$$\int_{0}^{\infty }C_{1}^{i}\;dt=\int_{0}^{\infty }C_{v}(t)\;dt\;\text{for}\;2\leq i\leq n.$$

Finally, for 1≤*i*≤*n*,

$$\begin{array}{rl} 0 & =\int_{0}^{\infty }\delta_{2}\frac{dM_{2}^{i}}{dt}+\delta_{2}\frac{dM_{3}^{i}}{dt}\;dt=\int_{0}^{\infty }a^{i}k_{1}(C_{1}^{i}-C_{2}^{i})\;dt\\ & \Rightarrow \int_{0}^{\infty }C_{2}^{i}\;dt=\int_{0}^{\infty }C_{1}^{i}\;dt=\int_{0}^{\infty }C_{v}(t)\;dt \end{array}$$

and

$$\begin{array}{rl} 0 & =\int_{0}^{\infty }\frac{dM_{3}^{i}}{dt}\;dt=\int_{0}^{\infty }V^{i}k_{2}C_{2}^{i}(C_{0}-C_{3}^{i})-V^{i}k_{-2}C_{3}^{i}\;dt\\ & \Rightarrow k_{-2}\int_{0}^{\infty }C_{3}^{i}\;dt+k_{2}\int_{0}^{\infty }C_{2}^{i}C_{3}^{i}\;dt=k_{2}C_{0}\int_{0}^{\infty }C_{v}(t)\;dt\\ & \Rightarrow \beta \int_{0}^{\infty }C_{3}^{i}\;dt+\int_{0}^{\infty }C_{2}^{i}C_{3}^{i}\;dt=C_{0}\int_{0}^{\infty }C_{v}(t)\;dt. \end{array}$$

It follows from this that

A 5 $$\frac{C_{0}-\max_{t}C_{3}^{i}}{\beta }\int_{0}^{\infty }C_{v}(t)\;dt\leq \int_{0}^{\infty }C_{3}^{i}\;dt\leq \frac{C_{0}}{\beta }\int_{0}^{\infty }C_{v}(t)\;dt.$$
