# Supplementary material for: Drug delivery in a tumour cord model: a computational simulation
Source: R Soc Open Sci. 2017 May 24;4(5):170014. doi: 10.1098/rsos.170014 (PMC5451806; doi:10.1098/rsos.170014)
Supplement: Additional profiles and model description [file rsos170014supp1.pdf]

1 Supplementary Material

Article Title: Drug delivery in a tumour cord model: a computational simulation
Authors: M.E.Hubbard, M.Jove, P.M.Loadman, R.M.Phillips, C.J.Twelves, S.W.Smye
Journal: Royal Society Open Science

1.1 Pharmacokinetic Profiles

The variations of concentration and exposure (integral of concentration with respect to time) in time are shown for each of the three pharmacokinetic supply profiles described in Section 3 of the main manuscript, in Figures 1 and 2, respectively. We acknowledge that profile PK3 is not physiologic, but instead represents a prolonged exposure at constant concentration.

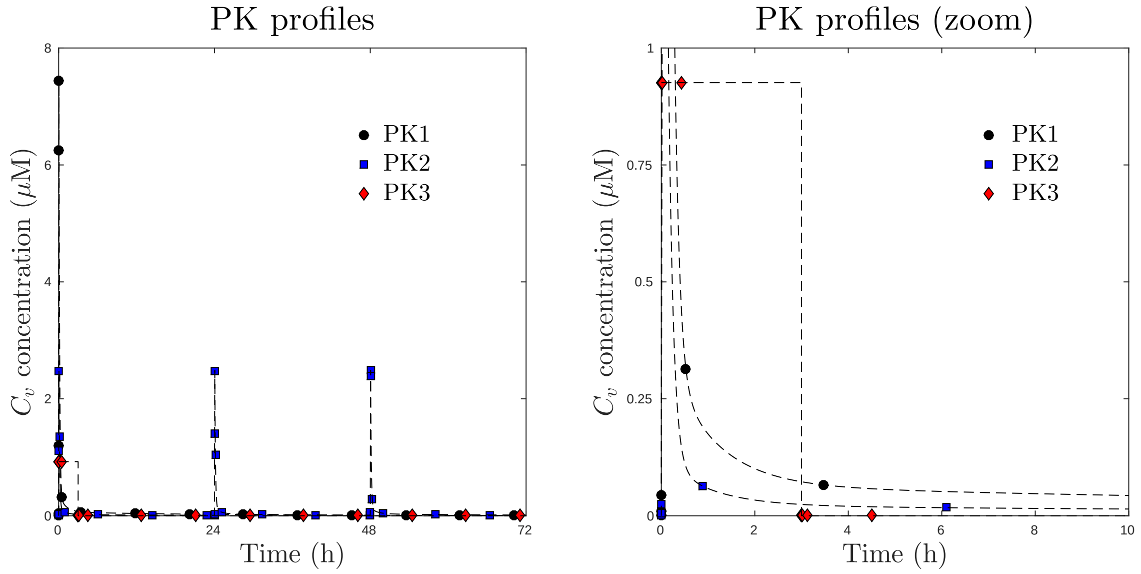

Figure 1: Comparison of concentration profiles, $C_v(t)$, for the three input PK profiles described in Section 3 of the main manuscript. The right-hand plot shows a magnification of the bottom left corner of the left-hand plot.

1.2 Two-Dimensional Profiles

The variations of concentration and exposure (integral of concentration with respect to time) in time are shown at points in the tissue close to and far from the source, for each of the three pharmacokinetic profiles described in Section 3 of the main manuscript, in Figures 3 (free extracellular drug, C_1) and 4 (bound intracellular drug, C_3).

- Close to the supply, the concentration and exposure profiles of the free extracellular drug (C_1) are very similar to those of the supplied PK profiles, $C_v(t)$, shown in Figures 1 and 2. At greater distances from the supply the shapes of the curves remain similar but the concentrations are lower.

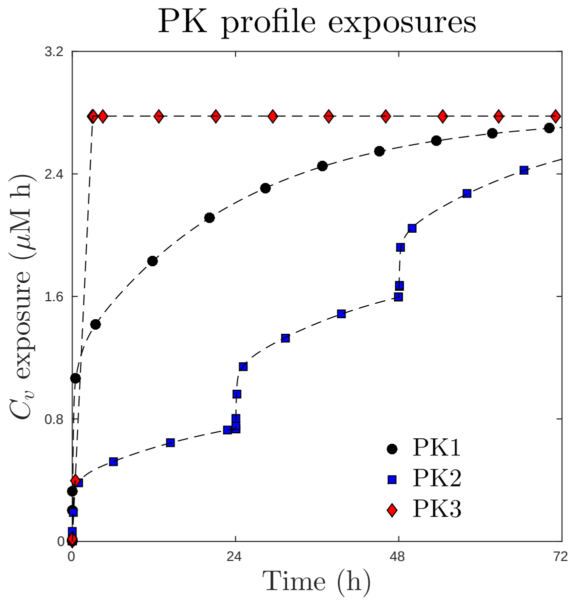

Figure 2: Comparison of exposure profiles, $(\int_0^t C_v(\tau)\ \mathrm{d}\tau)$, for the three input PK profiles described in Section 3 of the main manuscript.

- The bound intracellular drug profiles also have similarities, but they are much smoother. They take longer to reach peak concentrations and the decay from those concentrations is much slower. This is exaggerated further away from the supply.

Figure 5 illustrates one potential consequence of changing the supplied pharmacokinetic profile. A threshold is assigned and, if the exposure to bound intracellular drug within a computational compartment exceeds this value, the cells are assumed to die, otherwise they remain alive, as described in the remark at the end of Section 4.3. For the specified parameters (here chosen to represent narrow, leaky, vessels) and this threshold value, the uniform profile representing prolonged exposure at constant concentration, PK3, given by Equation (14) in Section 3 of the main manuscript, kills the most cells (shown on the right of the figure), while the profile representing three short infusions, PK2, given by Equation (13) in Section 3 of the main manuscript, kills the least (shown in the middle of the figure).

1.3 Spherically-Symmetric Compartment Model (1D)

In [1] the binding model was augmented with a spatial component by exploiting the shell-like nature of tumour cords, the geometric property that cells are broadly arranged in concentric circles around a central blood vessel. Here we consider a different geometry, made up of concentric spherical shells with no central vessel and drug being supplied at the outer boundary. This models an avascular multicell spheroid, bathed in drug. It is assumed that the rate of transport of drug between neighbouring shells is proportional to the shared interface

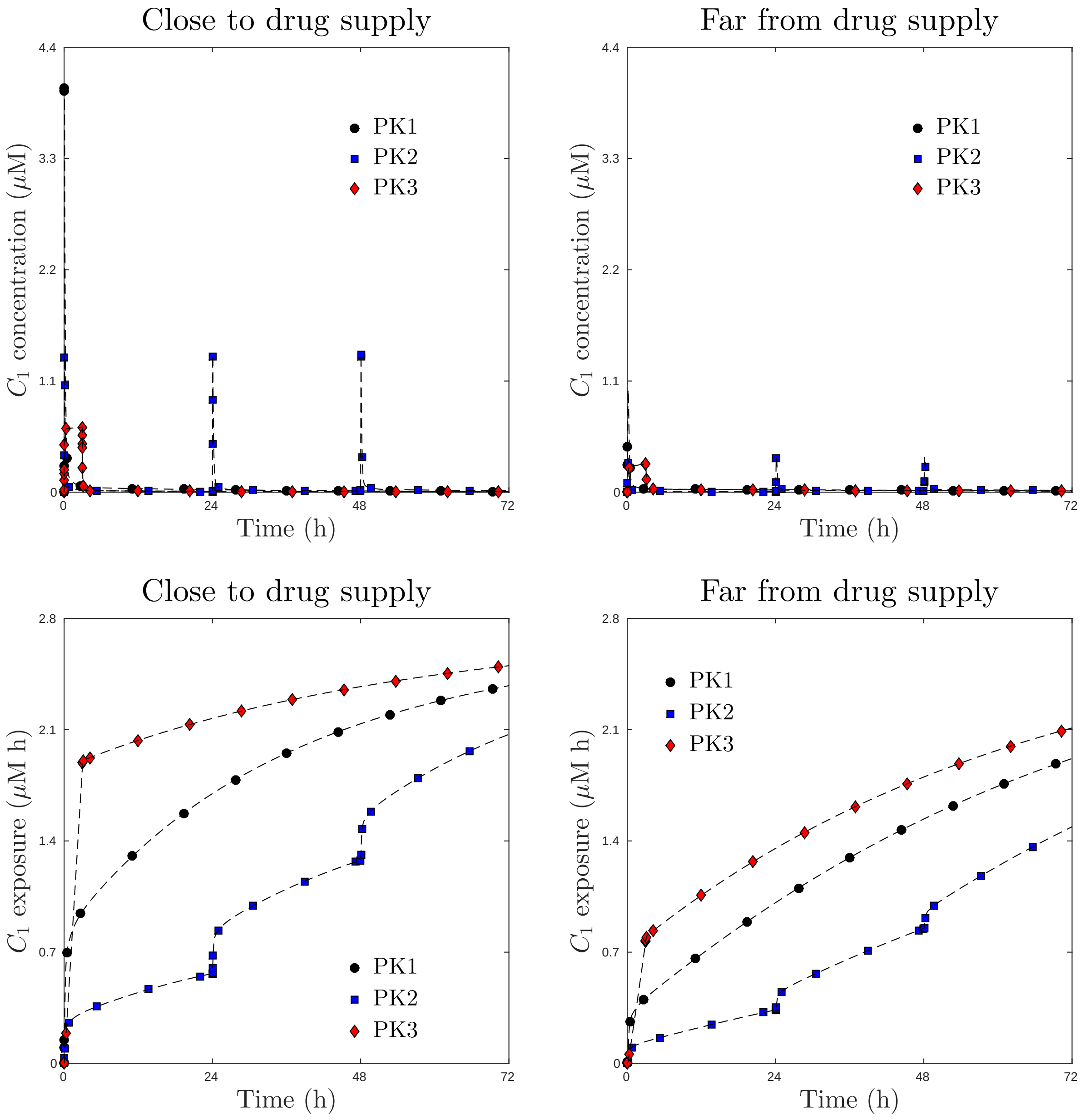

Figure 3: Dependence on time of the concentration of free extracellular drug (C_1, top) and exposure to free extracellular drug ($\int_0^t C_1(\tau)\,\mathrm{d}\tau$, bottom) at a point close to the supply of drug ($r = 26\,\mu$m, $z = 10\,\mu$m, left) and far from it ($r = 186\,\mu$m, $z = 490\,\mu$m, right).

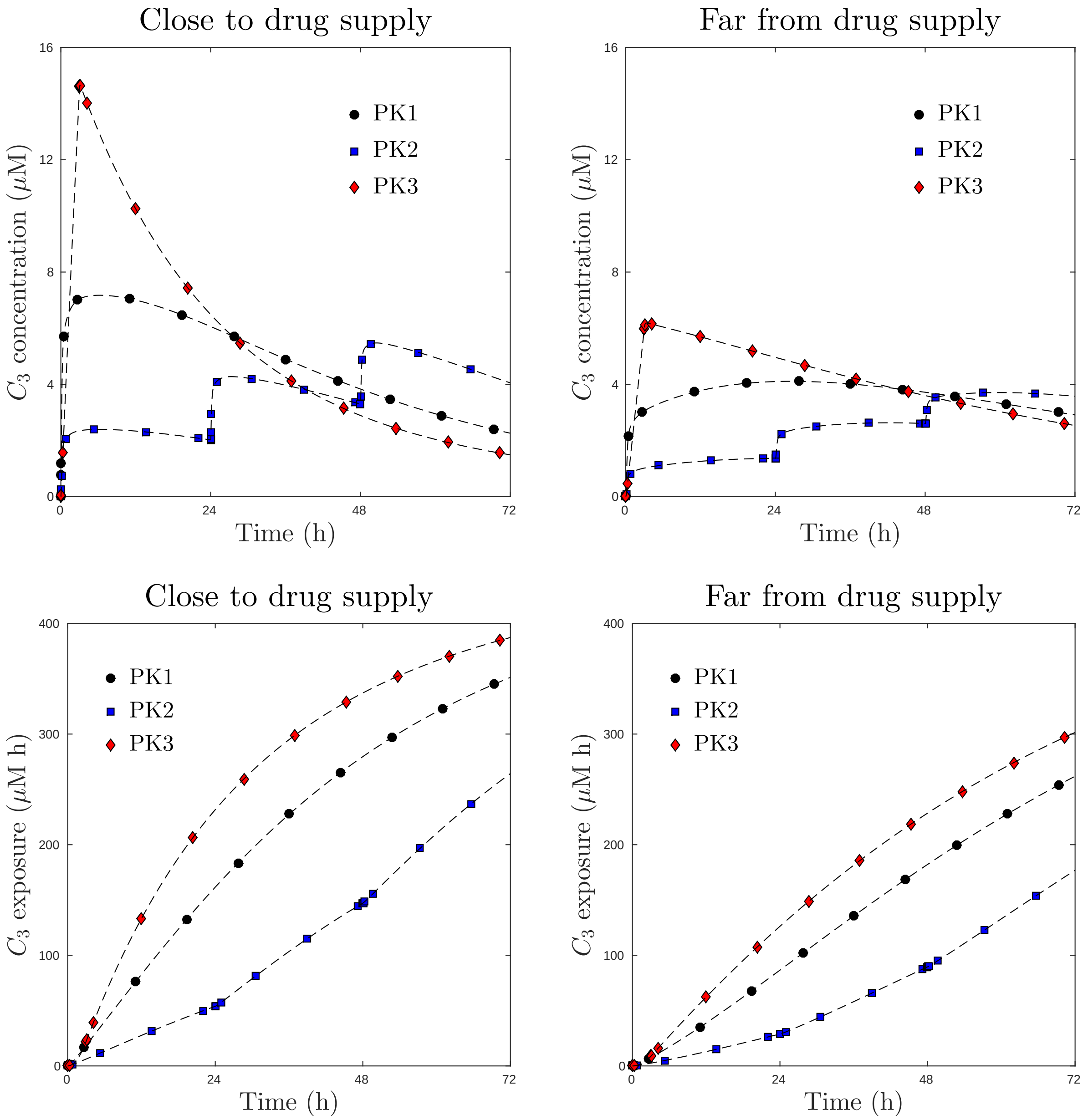

Figure 4: Dependence on time of the concentration of bound intracellular drug (C_3, top) and exposure to bound intracellular drug ($\int_0^t C_3(\tau)\,\mathrm{d}\tau$, bottom) at a point close to the supply of drug ($r = 26\,\mu$m, $z = 10\,\mu$m, left) and far from it ($r = 186\,\mu$m, $z = 490\,\mu$m, right).

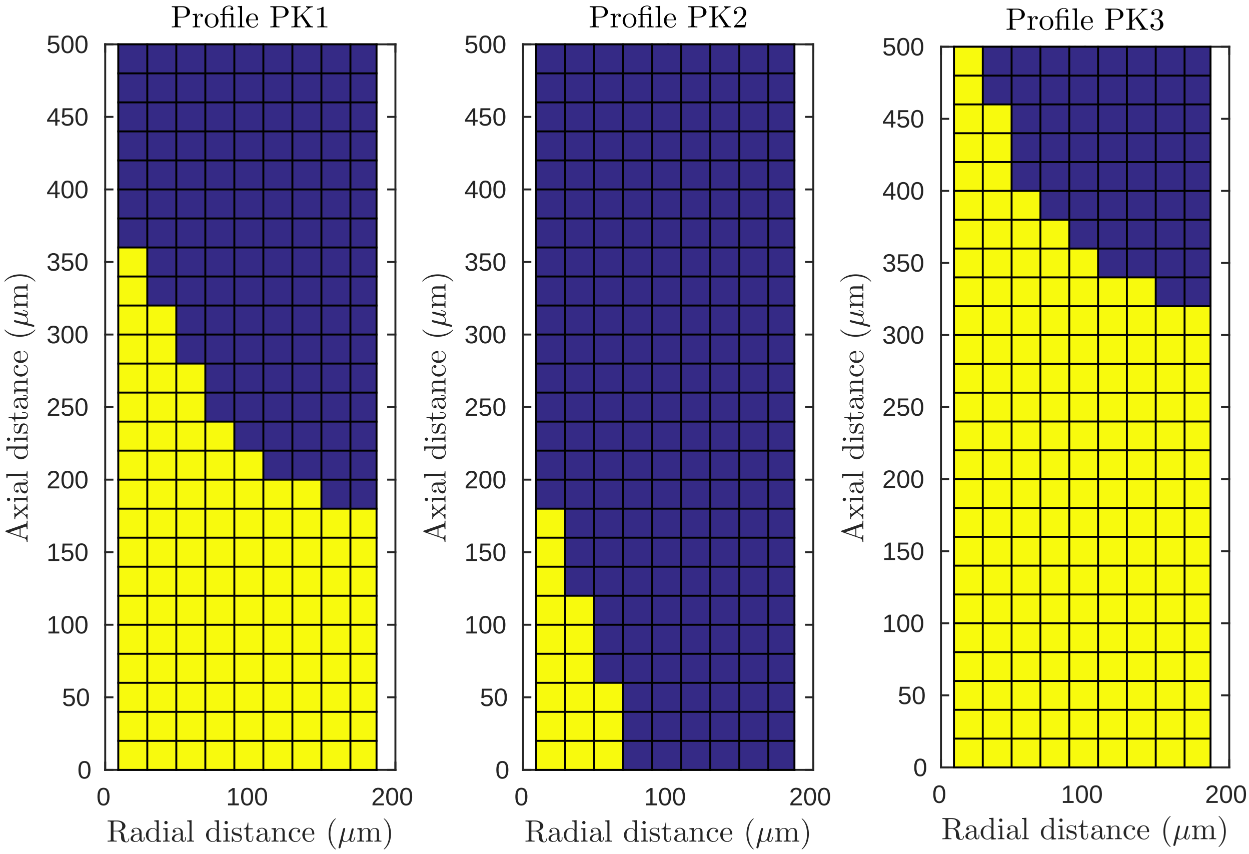

Figure 5: Thresholding of the exposure to bound drug ($\int_0^t C_3(\tau)\ \mathrm{d}\tau$) at $t = 72$ h: parameters as in Table 1 of the main manuscriptexcept that $k_v \to 10k_v$ (higher vessel wall permeability), $\lambda \to \lambda/10$ (lower blood flow velocity) and $l \to l/2$ (smaller vessel radius). Yellow (dead) cells have exposures above $165\,\mu$M h, blue (live) cells have exposures below this value.

area (denoted by $A^{i+1/2}$ for the interface between shells i and $i+1$) and the difference in concentration across the interface.

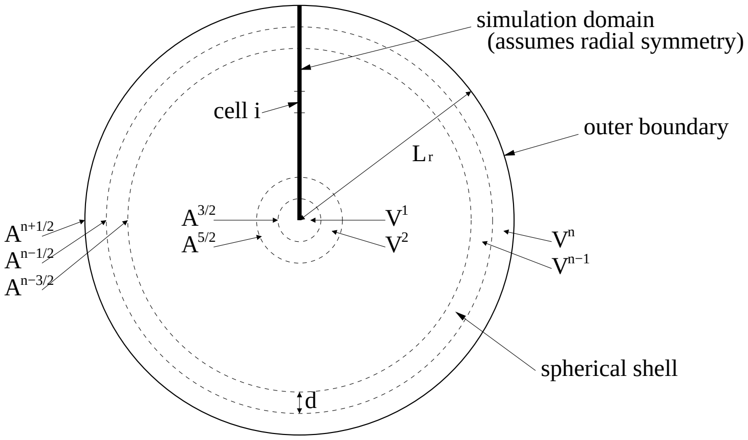

Figure 6: Slice through geometry for one-dimensional multicell spheroid compartment model with spherical symmetry and drug supplied from outside. The thick black vertical line represents the computational domain.

Under these assumptions, following the description of the one-dimensional cylindrically-symmetric compartment model developed in [1], the spatial variation in the radial direction can be included by augmenting Equations (1)-(3) of the main manuscript to give

$$\begin{aligned}\delta_1 V^i \frac{\mathrm{d}C_1^i}{\mathrm{d}t} &= A^{i-1/2}k_0(C_1^{i-1}-C_1^i) \;+\; A^{i+1/2}k_0(C_1^{i+1}-C_1^i) \\ &\qquad + a^i k_1(C_2^i - C_1^i)\,, \qquad (1)\\ \delta_2 V^i \frac{\mathrm{d}C_2^i}{\mathrm{d}t} &= a^i k_1(C_1^i - C_2^i) \\ &\qquad - \delta_2 V^i k_2 C_2^i (C_0 - C_3^i) \;+\; \delta_2 V^i k_{-2} C_3^i\,, \qquad (2)\\ \delta_2 V^i \frac{\mathrm{d}C_3^i}{\mathrm{d}t} &= \delta_2 V^i k_2 C_2^i (C_0 - C_3^i) \;-\; \delta_2 V^i k_{-2} C_3^i\,, \qquad (3)\end{aligned}$$

for $i = 1, \ldots, n$, where n is the number of shells, and a^i is the cellular surface area within the ith shell. The superscript corresponds to the shell number, and this index increases with distance from the centre of the spherical coordinates. Half-indices correspond to interfaces between shells, as illustrated in Figure 6.

These equations are precisely the same as those of our one-dimensional, cylindrically-symmetric, computational model (Equations (7)-(9) in [1]) – the model differs only in the definition of the geometric quantities and the boundary conditions. At the outer boundary, a predefined pharmacokinetic profile, $C_v(t)$, is prescribed, so when $i = n$ the term $A^{n+1/2}k_0(C_1^{n+1} - C_1^n)$ is replaced by $A^{n+1/2}k_0(C_v(t) - C_1^n)$ in Equation (1). A symmetry/no-flux condition is imposed at the centre of the spheroid by replacing the term $A^{1/2}k_0(C_1^0 - C_1^1)$ with zero when $i = 1$ in Equation (1). The volumes V^i of the shells are determined from the

geometry: assuming a shell thickness d, the volume of the ith shell is

$$V^i = \frac{4}{3}\pi d^3 (i^3 - (i-1)^3)\,. \tag{4}$$

The factors $\delta_1 = \frac{\delta}{1+\delta}$ and $\delta_2 = \frac{1}{1+\delta}$ are defined so that $\delta_1 V^i$ and $\delta_2 V^i$ are, respectively, the extracellular and intracellular volumes in the ith layer (δ being the ratio of extracellular to intracellular volume). The interface area between shells i and $i+1$ is

$$A^i = 4\pi d^2 i^2\,. \tag{5}$$

Values are given for the geometric and transport parameters in Table 1 of the main manuscript, except that

$$\alpha = a^i/V^i = 3/(r\sqrt[3]{1+\delta})\,. \tag{6}$$

References

[1] CM Groh, ME Hubbard, PF Jones, PM Loadman, N Periasamy, BD Sleeman, SW Smye, CJ Twelves, RM Phillips. Mathematical and computational models of drug transport in tumours. *J R Soc Interface*, 11(94):20131173, 2014.
